# Single-cell transcriptome profiling reveals immunological fitness of HIV long-term non-progressors

**DOI:** 10.1128/jvi.01597-25

**Published:** 2025-11-24

**Authors:** Minjuan Shi, Rongfeng Chen, Tongjin Wu, Yu Liao, Tongxue Qin, Ziyu Wen, Junqi Liu, Xiu Chen, Beibei Lu, Chunxing Yi, Li Ye, Hao Liang, Caijun Sun, Junjun Jiang

**Affiliations:** 1 School of Public Health (Shenzhen), Sun Yat-Sen University, Shenzhen, China; 2 Guangxi Key Laboratory of AIDS Prevention and Treatment & School of Public Health, Guangxi Medical University 74626, Nanning, Guangxi, China; 3 Jiont Laboratory for Emerging Infections Diseases in China (Guangxi)-ASEAN, Life Sciences Institute, Guangxi Medical University 666892, Nanning, Guangxi, China; 4 Hangzhou Institute of Medicine, Chinese Academy of Sciences, Hangzhou, Zhejiang, China; 5 Institute for Health Innovation and Technology, National University of Singapore 37580, Singapore, Singapore; 6 Key Laboratory of Tropical Disease Control (Sun Yat-Sen University), Ministry of Education 26469 , Guangzhou, China; 7 Shenzhen Key Laboratory of Pathogenic Microbes and Biosafety, Shenzhen Campus of Sun Yat-Sen University, Shenzhen, China; University Hospital Tübingen, Tübingen, Germany

**Keywords:** HIV reservoirs, long-term non-progressors, scRNA-seq, HIV cure

## Abstract

Long-term non-progressors (LTNPs) represent a valuable model to investigate immunological features associated with non-progression in chronic HIV infection. In this study, single-cell RNA sequencing was performed on peripheral blood mononuclear cells obtained from 12 individuals, including 4 LTNPs, 4 typical progressors (TPs), and 4 healthy donors (HDs). Compared with TPs, LTNPs exhibited a higher percentage of naive T cells, whereas TPs showed a higher percentage of CD8^+^ Effector-GNLY cells. LTNPs also exhibited lower gene-expression scores related to T-cell activation, cytotoxicity, inflammation, and interferon-alpha response across multiple effector-state T-cell subsets. GO enrichment analysis showed that compared with HDs and TPs, LTNPs exhibited downregulation of migration, translation, and antiviral response pathways in both CD4^+^ and CD8^+^ T cells, with upregulation of stress/inflammatory response and differentiation programs in CD4^+^ T cells and of immune differentiation/activation pathways in CD8^+^ T cells. High-dimensional weighted gene co-expression network analysis (hdWGCNA) further revealed higher module eigengene expression of innate and myeloid-related modules and lower expression of B-cell and cytotoxicity-related modules in LTNPs compared with TPs. Additionally, HIV-1 RNA^+^ cells were detected more frequently in TPs than in LTNPs and predominantly identified within CD4^+^ T cells. Together, these findings reveal a transcriptionally balanced and favorable immunological profile associated with non-progressive HIV infection and inform the design of immune-based strategies toward a functional HIV cure.

**IMPORTANCE:**

Understanding molecular traits associated with natural control in LTNPs is critical for advancing HIV remission and cure strategies. Using single-cell RNA sequencing, we found that LTNPs had more naive T cells and fewer CD8^+^ effector-GNLY cells than typical progressors, along with lower activation, cytotoxicity, inflammation, and IFN-α scores across effector-state T-cell subsets. Differential expression and GO analyses showed predominant downregulation of migration, translation, and antiviral pathways, while preserving early activation markers. Co-expression network analysis revealed higher innate/immune-regulatory module activity and lower B-cell and cytotoxicity modules in LTNPs. HIV-1 RNA^+^ cells were detected less frequently in LTNPs and mainly detected in CD4^+^ T cells. These findings highlight balanced immune programs in LTNPs, which may inform strategies toward a functional HIV cure.

## INTRODUCTION

Chronic HIV infection leads to the progressive depletion of CD4^+^ T cells and functional exhaustion of CD8^+^ T cells, ultimately resulting in acquired immunodeficiency syndrome (AIDS) (1–3). Although antiretroviral therapy (ART) durably suppresses ongoing viral replication and thus maintains plasma viral load at undetectable levels, it fails to eradicate the virus (4). Despite suppression, people living with HIV (PLWH) often exhibit residual immune activation and inflammation associated with long-term complications. HIV persistence during ART is maintained not only by long-lived latent reservoirs but also by multiple processes, including clonal expansion of infected cells, homeostatic proliferation, and antigen-driven proliferation (5–8). These processes allow the integrated provirus to persist despite effective viral suppression. Notably, approximately 1%–5% of untreated HIV-infected individuals, known as long-term non-progressors (LTNPs), remain ART naive for more than 7 years while maintaining stable CD4^+^ T cell counts (>500 cells/µL) and delayed immunologic decline (9–12), in contrast to typical progressors (TPs), who exhibit progressive immune depletion and disease progression (13). The distinct immunoregulatory features observed in LTNPs make them an important population for studying immune regulation in chronic HIV infection.

Although several factors have been implicated in the ability of LTNPs to control HIV, the exact mechanisms remain incompletely defined. For example, the CCR5Δ32 mutation confers resistance to HIV entry by impairing the CCR5 co-receptor (14), and protective HLA alleles such as HLA-B57 and HLA-B27 are associated with enhanced cytotoxic T-lymphocyte responses and improved control of viremia (15). Some LTNPs may also harbor attenuated viral strains or mutations in viral regulatory genes that reduce viral replication (16), and HIV integration into regions of low transcriptional activity may further limit viral expression and promote long-term persistence (17). However, many LTNPs lack these factors, suggesting that additional immune processes may contribute. T cells, particularly CD4^+^ and CD8^+^ T cells, are critical for viral control, but the dynamics of T-cell subsets in LTNPs remain unclear. One of the major barriers to curing HIV infection is the latent reservoir in resting memory CD4^+^ T cells, making it crucial to identify features that enrich for these infected cells crucial for reservoir-targeted strategies (18). Markers such as CD32a, CD73, CD161, and SERPINB9 have been reported to enrich for latently infected or reservoir-enriched CD4^+^ T cells (19–22). However, no single marker exclusively identifies HIV-infected cells, likely reflecting the heterogeneity of activation state, memory differentiation, and exhaustion phenotypes (23–26).

Single-cell RNA sequencing (scRNA-seq) has become a valuable tool for studying HIV infection, enabling high-resolution analysis of immune cell characteristics and transcriptional profiles (27–30). To explore the features associated with non-progression, we performed scRNA-seq to examine the immune profiles and transcriptional programs of T-cell subsets in LTNPs and TPs, focusing on gene-expression patterns related to immune activation and cytotoxicity, as well as pathways linked to antiviral responses. Our study aimed to determine whether LTNPs exhibit distinct transcriptomic features of immune regulation. By generating a high-resolution transcriptional map of T cells, we aimed to define transcriptomic correlates of immunological non-progression in LTNPs and generate hypotheses that may inform future mechanistic studies and guide the design of immune-based interventions toward a functional HIV cure.

## RESULTS

### Analysis of PBMC composition in HIV-infected individuals

**Fig 1:**
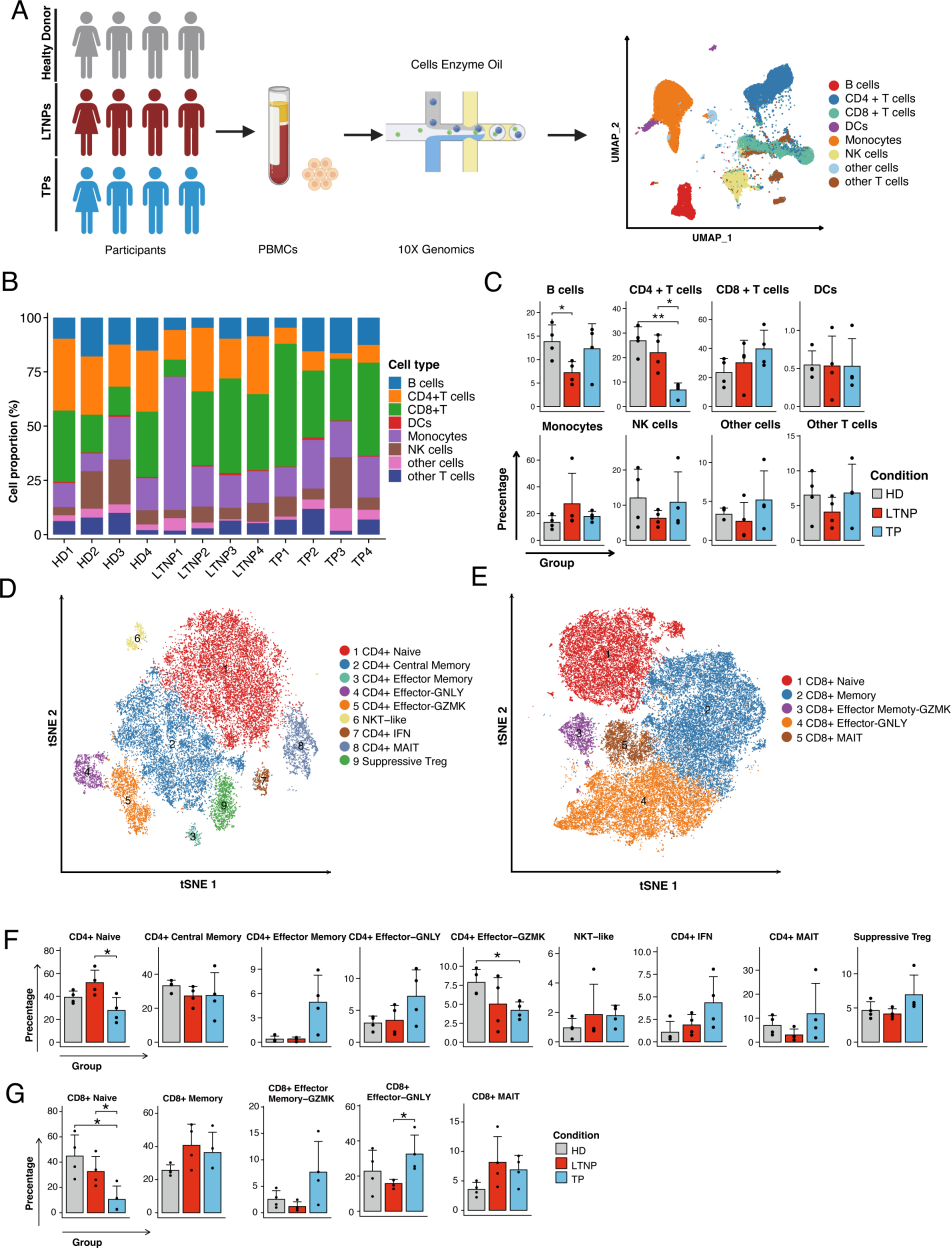
Compositional analysis of main cell types among LTNPs, TPs, and HDs. (**A**) Schematic illustrating the overall study design and the clustering of PBMCs generated by multimodal reference mapping. (**B-C**) The distribution of different cell subsets across samples, with the x-axis representing different samples and the y-axis representing percent composition. (**B**) Condition preference of each cluster. Y-axis: average percent composition across the three conditions. Conditions are shown in different colors. (**C**) Each bar plot represents one cell cluster. Error bars represent ± SEM. for 4 healthy donors, 4 LTNPs, and 4 TPs. * *P* < 0·05, two-sided unpaired *t*-test. (**D-E**) Cellular populations identified for CD4^+^ (**D**) and CD8^+^ (**E**) T cells. The t-SNE projections show 9 CD4^+^ and 5 CD8^+^ clusters derived from HD (*n* = 4), LTNP (*n* = 4), and TP (*n* = 4) samples, with each dot representing a single cell and colored according to cell types. Minor subsets with very few cells were excluded from downstream analyses (see Fig. S2 for complete clustering). (**F-G**) Percentages of T-cell subsets across conditions for CD4^+^ (**F**) and CD8^+^ (**G**) T cells. Y-axis: average percent of samples across the three conditions. Conditions are shown in different colors. Each bar plot represents one cell cluster. Error bars represent ± SEM for 4 healthy donors and 8 HIV-1-infected individuals. *P < 0·05; two-sided unpaired *t*-test.

Droplet-based scRNA-seq (10 × Genomics) was performed on PBMCs from LTNPs, TPs, and healthy donors (*n* = 4 each). Longitudinal pre-ART CD4^+^ T-cell counts for LTNPs and TPs are shown in Fig. S1, and the characteristics of the 12 participants are summarized in Table S1. In total, we analyzed 159,493 PBMC transcriptomes. After SCTransform normalization, multimodal reference mapping defined eight major cell clusters (Fig. 1A ; Fig. S2 ) (31). Per-donor compositions (Fig. 1B) and group-wise summaries (Fig. 1C) showed lower B-cell proportions in LTNPs than HDs and higher CD4^+^ T-cell proportions in LTNPs than in TPs, whereas other major subsets, including CD8^+^ T cells, monocytes, and NK cells, showed no significant differences across groups. At the selected resolution, graph-based unsupervised clustering on PCA/Harmony embeddings initially resolved 13 CD4^+^ and 9 CD8^+^ subpopulations (Fig. S2). Because several minor subsets contained very few cells, these were excluded from downstream analyses, resulting in 9 CD4^+^ and 5 CD8^+^ clusters that were retained and visualized using t-SNE (Fig. 1D and E). Cluster annotation was based on canonical marker genes, with representative marker expression profiles shown in Fig. S3. Per-donor counts of major immune subsets and T-cell subpopulations are summarized in Table S2.

We next examined T-cell heterogeneity across groups. Among CD4^+^ T cells, LTNPs exhibited a significantly higher percentage of CD4^+^ naive T cells than TPs. TPs also showed a lower percentage of CD4^+^ Effector-GZMK cells than HDs (Fig. 1F). In the CD8^+^ compartment, the percentage of CD8^+^ naive T cells was reduced in TPs compared with both LTNPs and HDs. Consistent with previous reports, LTNPs showed lower levels of CD8^+^ effector-GNLY cells, a subset often associated with poor immune restoration (Fig. 1G)(30).

### Transcriptomic changes of CD4^+^ T cell subsets across disease conditions

**Fig 2:**
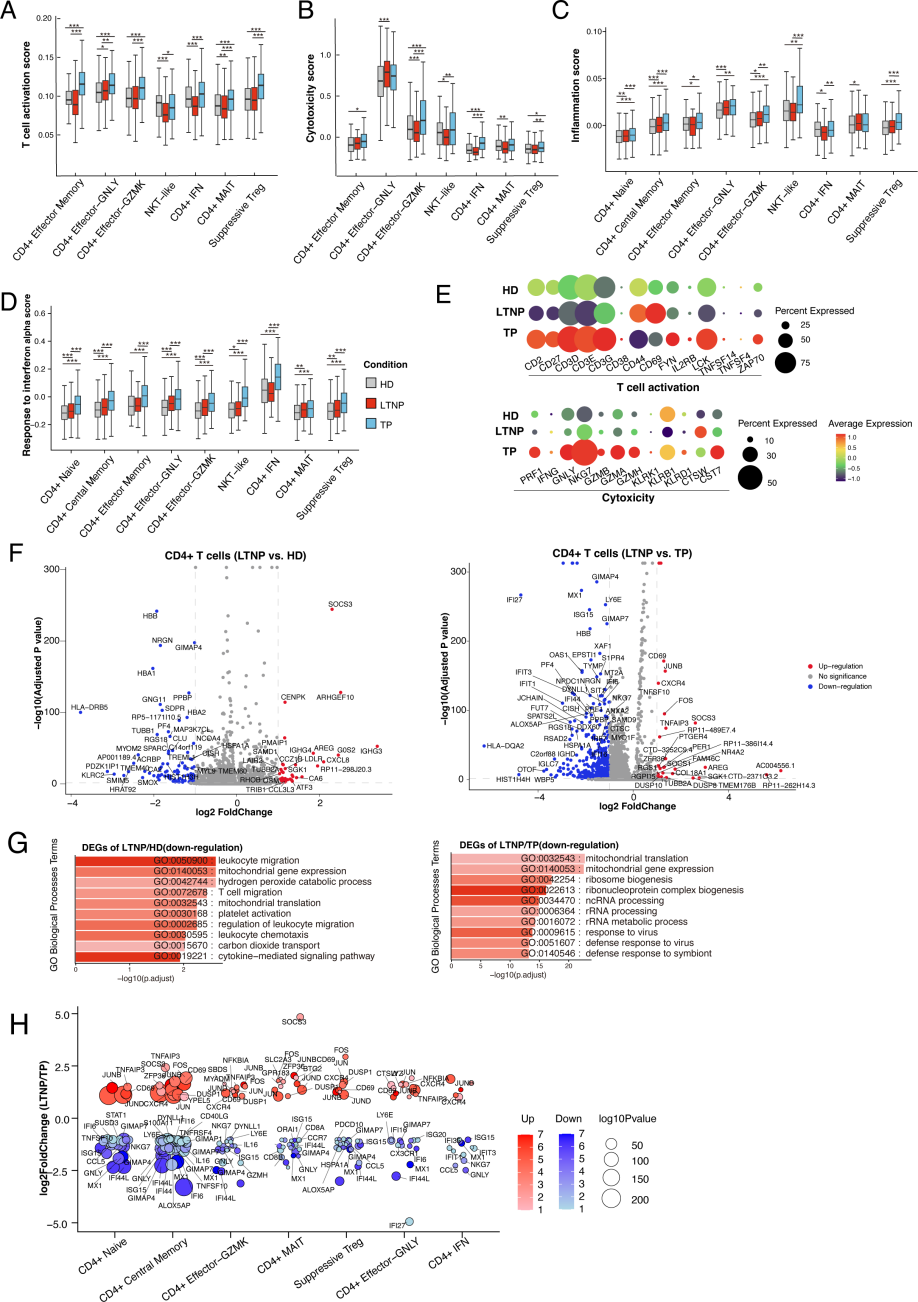
Immunological profiling of CD4^+^ T subsets. (**A–D**) Box plots of T-cell activation (**A**), cytotoxicity (**B**), inflammation (**C**), and IFN-α response (**D**) scores across clusters and conditions. Horizontal lines represent median values, with whiskers extending to the farthest data point within a maximum of 1.5 × interquartile range. * *P* < 0·01; ** *P* < 0·001; *** *P* < 0·0001; two-sided unpaired *t*-test (Outliers outside the box were removed). (**E**) Dot plot showing expression of some genes associated with T cell activation and cytotoxicity scores across the three conditions. The size of the circle indicates the percentage of cells expressing pathway-associated genes under each condition. The color of the circle represents the expression levels of pathway-associated genes under each condition. (**F**) Volcano plot of DEGs in CD4^+^ T cells comparing LTNP vs. HD (left panel) and LTNP vs. TP (right panel). The Wilcoxon rank-sum test was used. Genes with *adjusted P* ≤ 0.05 and average log₂-fold change ≥ 0.25 are shown as significant. (**G**) Gene enrichment analysis of the downregulated DEGs identified in (**F**). GO terms are labeled with name and ID and sorted by −log10 ***P*** value. A brighter color indicates a higher number of genes. The top 10 enriched GO terms are shown with *adjusted P* value ≤ 0.05 and average log2(fold change) ≥ 0.5. (**H**) Log2-fold change (y-axis) of DEGs between LTNPs and TPs in the CD4^+^ T-cell subset. Red and blue indicate genes that are upregulated and downregulated in LTNPs, respectively. Point size represents statistical significance (*adjusted P* value), and transparency denotes the number of comparisons in which each gene was significantly differentially expressed. Low-count subsets were excluded during reanalysis to enhance statistical robustness.

Differential expression analyses were performed for CD4^+^ T-cell subsets with significant percentage differences across groups (Fig. 1F). LTNP naive CD4^+^ T cells showed reduced expression of interferon-stimulated genes compared with TPs, whereas TP effector-GZMK cells upregulated interferon and cytotoxic genes relative to HDs (Fig. S4A). We next calculated cytotoxicity, T-cell activation, inflammation, and interferon-alpha scores for effector-state CD4^+^ T-cell subsets across HDs, LTNPs, and TPs to compare their transcriptional characteristics based on gene expression (hereafter, “score” denotes a gene-set expression score, not a direct functional assay). In LTNPs, several effector-state CD4^+^ T-cell subsets showed lower gene-expression-derived cytotoxicity scores than TPs, including CD4^+^ Effector-GZMK, NKT-like, and CD4^+^ IFN subsets (Fig. 2A). LTNPs also exhibited reduced T-cell activation, inflammatory, and IFN-α responses (Fig. 2B through D), supported by associated gene expression (Fig. 2E; Fig. S4B).

Differential expression analysis revealed fewer upregulated and more downregulated genes in LTNPs (Fig. 2F). Compared with HDs, the downregulated DEGs were enriched in pathways related to leukocyte migration, T-cell migration, mitochondrial gene expression and translation, and cytokine-mediated signaling (Fig. 2F and G; Fig. S4C). Compared with TPs, the downregulated genes were involved in ribosome biogenesis, rRNA processing and metabolism, ncRNA processing, and mitochondrial translation and gene expression, as well as response to virus infection (Fig. 2F and G; Fig. S4C). Across multiple CD4^+^ T-cell subsets, LTNPs expressed higher levels of early activation-related markers (JUNB, JUND, and CD69) but lower levels of interferon-responsive genes (IFI44L, ISG15, and MX1) compared with TPs (Fig. 2H). Although overall gene-set scores indicated reduced activation and inflammation in LTNPs, the increased expression of these early activation markers suggests preserved activation potential. In addition, downregulation of GIMAP4 and GIMAP7, together with reduced expression of exhaustion-associated genes, is consistent with a restrained yet competent activation state (Fig. 2H; Fig. S4D) (32). These associations are descriptive and based on gene expression and do not establish a mechanism.

### Transcriptomic profiles of CD8^+^ T cell subsets across disease progression

**Fig 3:**
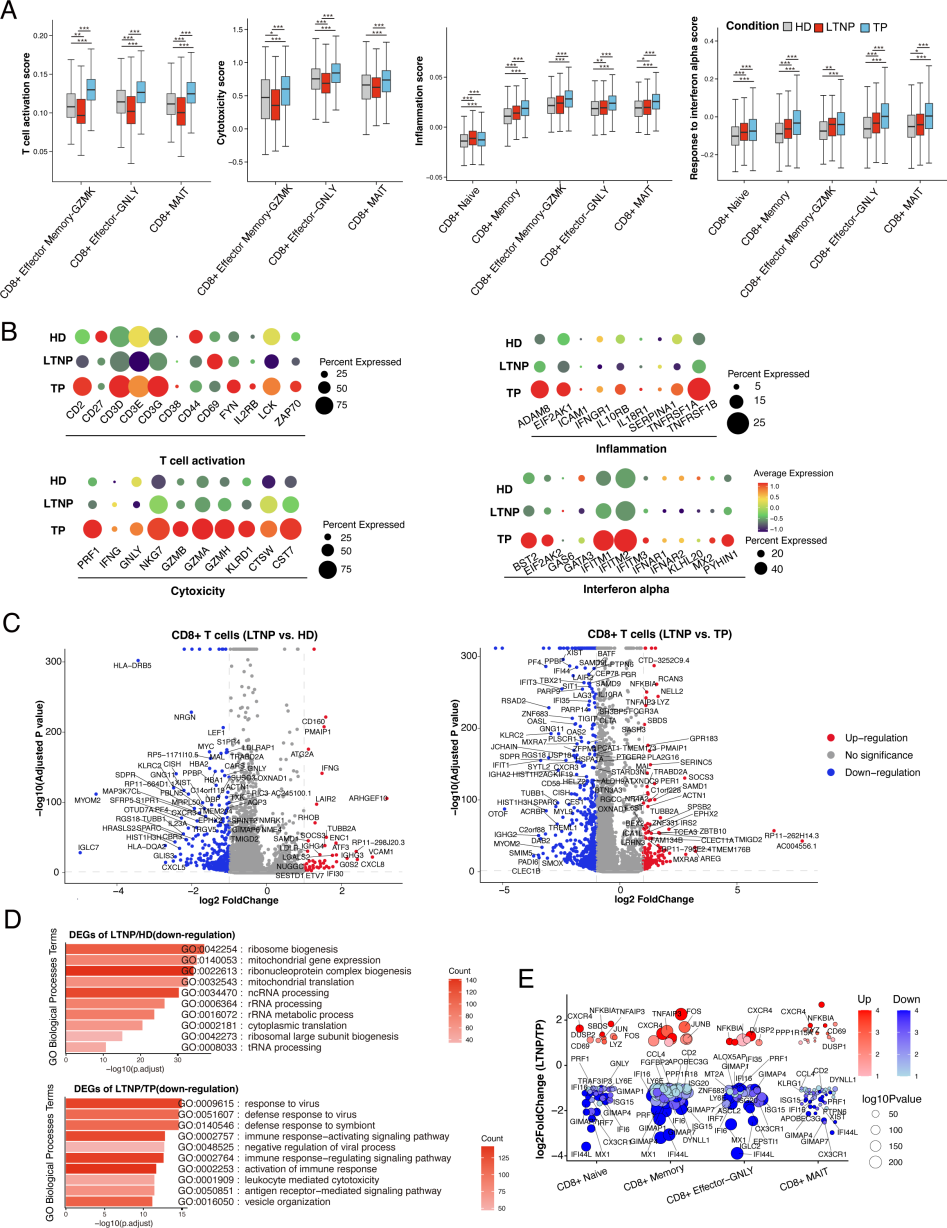
Immunological characterization of CD8^+^ T cell subsets. (**A**) Box plots of T-cell activation, cytotoxicity, inflammation, and IFN-α response scores across clusters and conditions. Horizontal lines represent median values, with whiskers extending to the farthest data point within a maximum of 1.5 × interquartile range. *P < 0·01; **P < 0·001; ***P < 0·0001; two-sided unpaired *t*-test (Outliers outside the box were removed). (**B**) Dot plot showing expression of pathway-associated genes corresponding to the scores in (**A**) across conditions. Dot size indicates the percentage of expressing cells; color represents the expression level. (**C**) Volcano plot of DEGs in CD8^+^ T cells comparing LTNP vs. HD (left panel) and LTNP vs. TP (right panel). The Wilcoxon rank-sum test was used. Genes with *adjusted P* ≤ 0.05 and average log₂-fold change ≥ 0.25 are shown as significant. (**D**) Go enrichment analysis of the downregulation DEGs identified in (**C**). GO terms are labeled with name and ID and sorted by −log10 ***P*** value. A brighter color indicates a higher number of genes. The top 10 enriched GO terms are shown with *adjusted P* ≤ 0.05 and average log2(fold change)≥0.5. (**E**) Log2-fold change (y axis) of DEGs between LTNP and TP individuals in the CD4^+^ T cell subset. Red and blue indicate genes that are upregulated and downregulated in LTNPs, respectively. Point size represents statistical significance (*adjusted P* value), and transparency denotes the number of comparisons in which each gene was significantly differentially expressed. Low-count subsets were excluded during reanalysis to enhance statistical robustness.

Differential expression analyses were performed for CD8^+^ T-cell subsets with significant percentage differences across groups (Fig. 1G). TP-naive CD8^+^ T cells upregulated interferon-stimulated and activation-related genes compared with HDs, whereas LTNPs showed lower expression of interferon and cytotoxic genes in both naive and effector-GNLY subsets relative to TPs (Fig. S5A). CD8^+^ T-cell subsets from LTNPs generally exhibited lower T-cell activation and cytotoxicity scores than those from TPs, including CD8^+^ Effector Memory-GZMK, CD8^+^ Effector-GNLY, and CD8^+^ MAIT cells (Fig. 3A). Most CD8^+^ T-cell subsets in LTNPs also showed reduced inflammatory and IFN-α response scores compared with TPs (Fig. 3A), supported by associated gene expression (Fig. 3B). To better understand the basis of these score differences, we next analyzed DEGs in CD8^+^ T-cell subsets. Similar to CD4^+^ T cells, the majority of DEGs in LTNP CD8^+^ T cells were downregulated (Fig. 3C). In LTNPs versus HDs, these downregulated DEGs were enriched in translational and ribonucleoprotein biogenesis pathways, including ribosome biogenesis, cytoplasmic and mitochondrial translation and gene expression, and rRNA/ncRNA/tRNA processing, indicating broad attenuation of protein synthesis (Fig. 3D). In LTNPs versus TPs, downregulated DEGs were enriched in antiviral and immune-activation pathways, such as responses to virus and symbiont stimuli, immune response–activating/regulating signaling, leukocyte-mediated cytotoxicity, and antigen receptor signaling (Fig. 3D). By contrast, upregulated DEGs in both comparisons were mainly related to lymphocyte activation, differentiation, and antigen presentation (Fig. S5B). Consistently, compared with LTNPs, TPs showed higher expression of exhaustion markers (LAG3, TIGIT) (Fig. S5C) and interferon-stimulated genes (MX1, IFI44L) (Fig. 3E), indicating a transcriptional profile marked by greater exhaustion and interferon-driven activation. Taken together, these transcriptomic patterns suggest a global immune-quiescent state across both CD4^+^ and CD8^+^ T cells in LTNPs, contrasted with heightened inflammatory and antiviral signatures in TPs.

### Identification of HIV RNA^+^ cells and differential gene expression profiles

**Fig 4:**
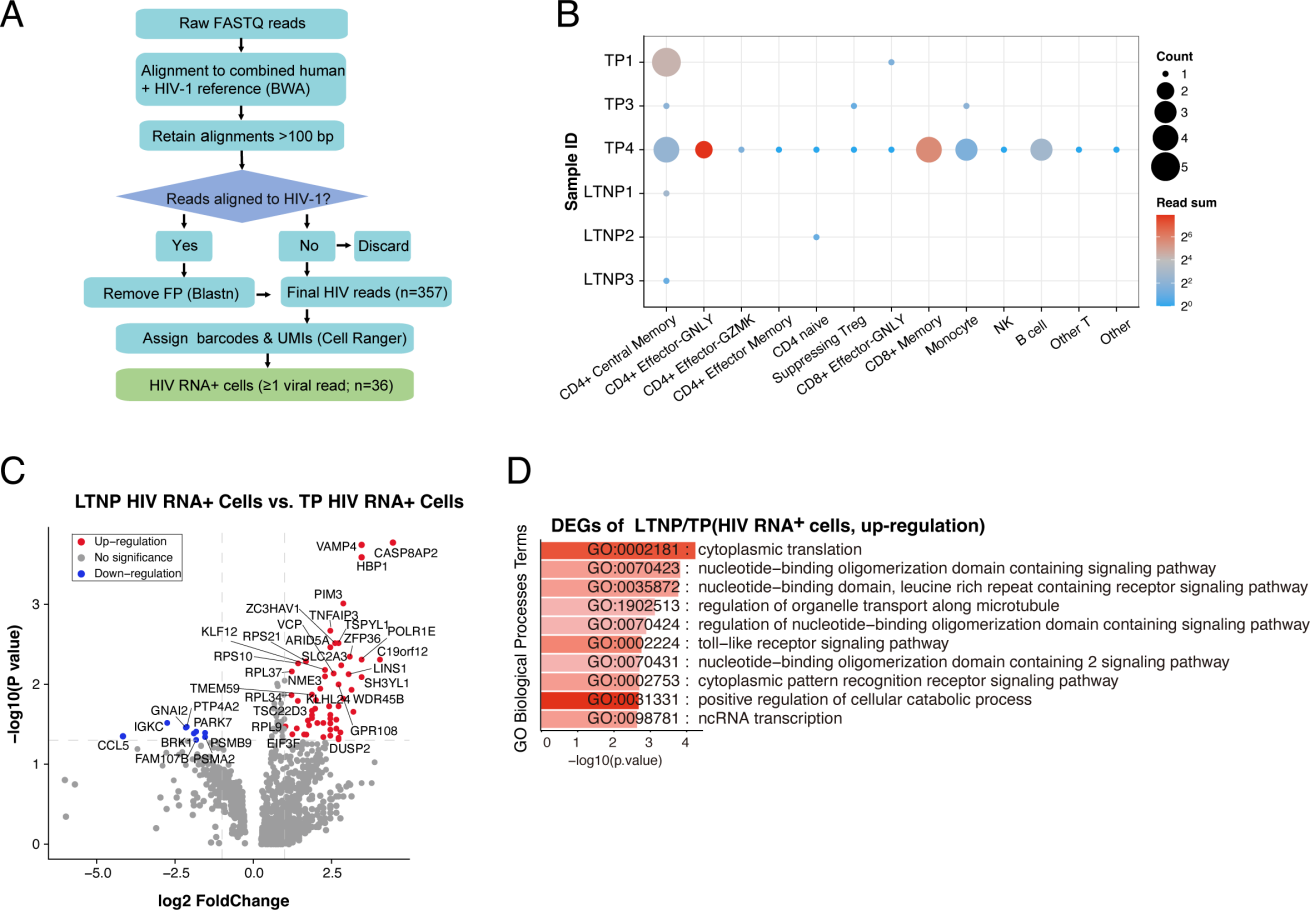
Transcriptomic profiling of HIV RNA^+^ cells. (**A**) Workflow to identify HIV RNA^+^ cells. Abbreviation: FP, false positives. (**B**) Distribution of cell subsets containing the 36 HIV RNA^+^ cells identified across all PBMCs. (**C**) DEGs between LTNP and TP HIV RNA^+^ cells, identified using the Wilcoxon test (*P* ≤ 0.05 and average log2(fold change) ≥0.25. (**D**) GO terms are labeled with name and id and sorted by −log10 *P* value. A brighter color indicates a higher number of genes. The top 10 enriched GO terms of the upregulated DEGs in (**C**) are shown with adjusted *P* value ≤ 0.05 and average log2(fold change) ≥0.5.

Our single-cell RNA sequencing (scRNA-seq) pipeline used the 10× Genomics 3′ Gene Expression assay, which captures host cellular and viral transcripts by reverse-transcribing polyadenylated mRNAs. Since HIV-infected cells contain polyadenylated HIV mRNAs, we screened PBMC transcriptomes by aligning reads to a combined human (GRCh38) and HIV-1 reference (Fig. 4A). We detected a small fraction of HIV RNA^+^ cells in 6 of 8 PLWH and none in uninfected controls (HDs). In total, 36 of 159,493 cells (0.023%) were HIV RNA^+^ (Table S3), consistent with prior 10× PBMC scRNA-seq studies reporting rare detection of HIV transcripts in blood without targeted enrichment (33, 34). Of these 36 cells, three were from LTNPs and 33 from TPs. In LTNPs, HIV RNA^+^ cells localized to CD4^+^ central memory and CD4^+^ naive subsets (Fig. 4B; Table S3). In TPs, HIV RNA^+^ cells were distributed across multiple populations: 6 cells in TP1 (5 CD4^+^ central memory cells and 1 CD8^+^ effector-GNLY cell), 3 in TP3 (1 CD4^+^ central memory cell, 1 suppressive Treg cell, and 1 monocytes), and 24 in TP4 (distributed across CD4^+^ and CD8^+^ T cells, B cells, monocytes and other T-cell subsets) (Fig. 4B; Table S3). TP1 and TP3 showed higher total viral UMI counts in the scRNA-seq libraries (Table S3). These counts reflect captured viral transcripts and are not a quantitative proxy for plasma HIV-1 RNA. Overall, 18 of 36 HIV RNA^+^ cells were CD4^+^ T cells.

Transcriptomic profiling of HIV RNA^+^ cells suggested distinct gene expression patterns and pathway enrichment between LTNPs and TPs (Fig. 4C and D). In LTNP HIV RNA^+^ cells, we observed relative upregulation of genes involved in cytoplasmic translation (e.g., RPL37, RPL34, and RPL9) and cellular catabolic processes, together with higher expression of nucleic acid-sensing-related genes (e.g., ZC3HAV1 and GPR108) (Fig. 4C and D). These observations may reflect higher expression of genes related to translation and innate sensing. In contrast, TP HIV RNA^+^ cells displayed gene expression patterns associated with immune activation and cellular stress responses (Fig. 4C and D). These findings are descriptive and do not imply a causal relationship with infection or disease activity. Cautiously, the HIV RNA^+^ cells identified in this study could represent only a small subset of transcriptionally active infected cells captured during sequencing, and the absence (e.g., in TP2) or low number of detectable RNA^+^ cells in infected patients likely reflects sampling stochasticity and technical sensitivity limits (35).

### HdWGCNA algorithm identifies key modules

**Fig 5:**
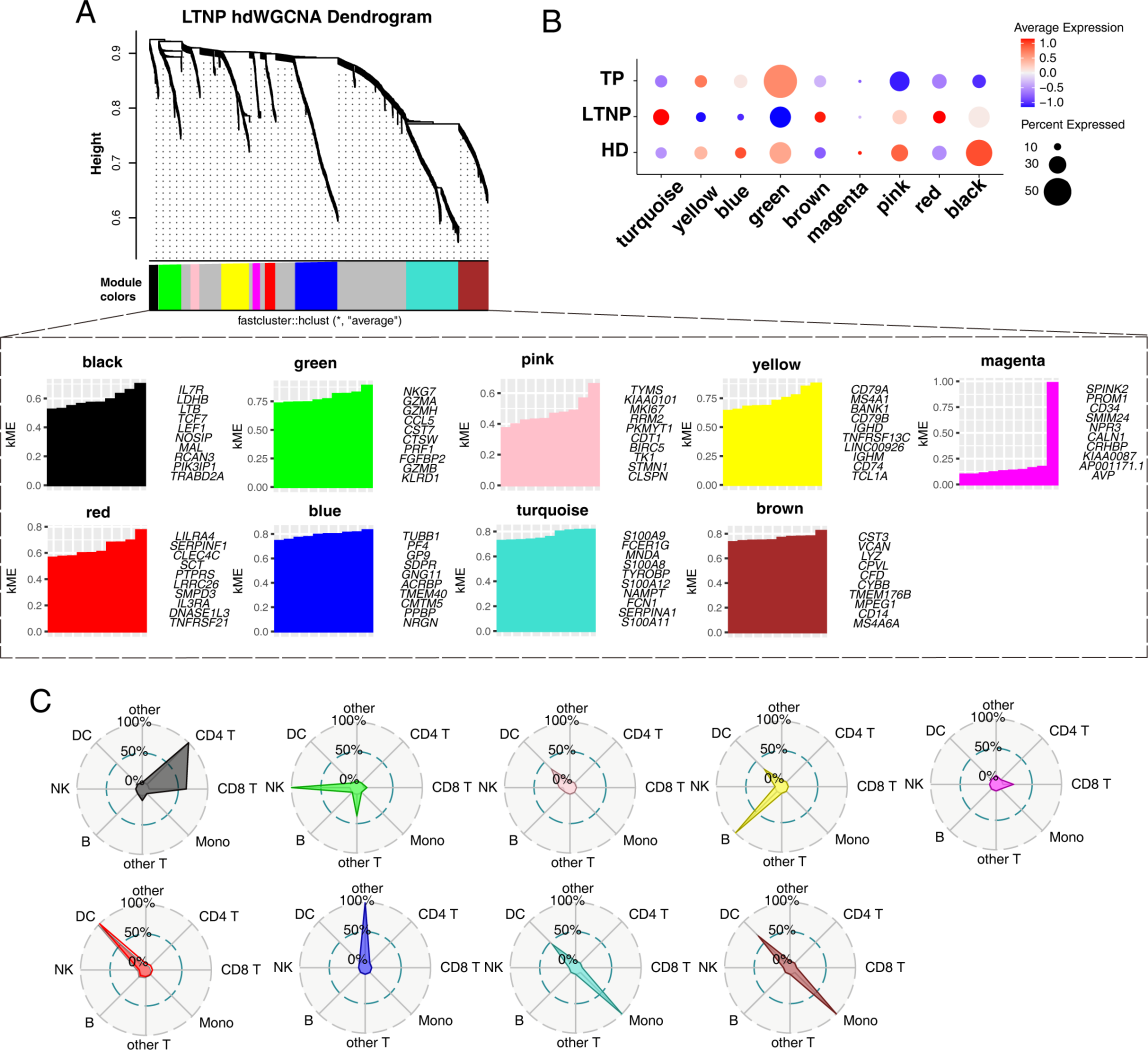
Single-cell WGCNA. (**A**) Construct a co-expression network using the optimal soft threshold of 4 and divide the genes into distinct modules to generate a gene clustering tree, with different colors representing different modules. The kME (Eigengene-based connectivity) map shows the top 10 hub genes in each module ranked by kME. Genes with higher connectivity or kME values are considered more central within their respective modules. (**B**) Dot plot presenting the average expression of module-specific hub genes in different groups, calculated by gene scoring for the top 25 hub genes ranked by kME in each module, color indicates average expression, size indicates percent expressed. (**C**) Radar plots showing the distribution of module-associated genes signatures across distinct immune cell types, including CD4^+^ T cells, CD8^+^ T cells, B cells, monocytes (Mono), dendritic cells (DCs), NK cells, and others. Each plot highlights the primary immune cell groups contributing to the expression of genes within each module.

High-dimensional WGCNA (hdWGCNA) of the integrated scRNA-seq data set (soft-threshold power = 4; Fig. S6A) identified nine co-expression modules (Fig. 5A). The top 10 hub genes per module, ranked by eigengene connectivity (kME), are shown in Fig. 5A, and genes with higher kME values are considered more central within their respective modules (Fig. S6B). Module eigengene score profiles clearly distinguished LTNPs, TPs, and HDs, with LTNPs characterized by higher turquoise and brown scores and lower yellow, blue, and green scores (Fig. 5B). Notably, turquoise and brown eigengenes were strongly positively correlated (Fig. S6C). These patterns indicate that LTNPs engage distinct module-level co-expression programs relative to the other groups. The cellular distributions of these module signatures, visualized by radar plots (Fig. 5C), were consistent with the GO enrichment of the top hub genes. The turquoise module was associated with innate immune signaling and antiviral defense, and the brown module with immune regulation and cytokine/chemokine production. In contrast, yellow (B-cell activation and antibody-mediated immunity) and green (NK-cell cytotoxicity and T-cell activation) were reduced in LTNPs. Additional modules included platelet activation and hemostasis (blue), cell cycle and chromosome segregation (pink), T-cell activation and adhesion (black), steroid response and organ development (magenta), and neurodevelopment, membrane potential, and ion transport (red). The red module, although annotated with neurodevelopmental terms, was confined to myeloid populations and should be interpreted with caution. A complete list of enriched terms is provided in Fig. S7. Collectively, these module-level associations highlight transcriptional programs that distinguish LTNPs from TPs and HDs, and provide a framework for future mechanistic studies.

## DISCUSSION

To date, neither a curative therapy nor a preventive vaccine exists for HIV, primarily due to an incomplete understanding of the immune correlates of protection (36, 37). Previous studies have profiled T-cell features in chronic HIV infection (2, 38, 39), including single-cell descriptions of T-cell transcriptional landscapes and TCR repertoires under antiretroviral therapy (30). However, systematic subset-level contrasts between LTNPs and TPs have been limited. In this study, we analyzed ART-naive LTNPs, TPs, and healthy donors using a multi-tiered framework combining gene expression–derived scores, pathway enrichment, hdWGCNA-based co-expression network analysis, and comparative assessment of HIV RNA^+^ cells. LTNPs were characterized by preserved naive T-cell pools and lower activation, cytotoxicity, inflammation, and interferon-α scores across multiple effector subsets. At the transcriptomic level, LTNPs exhibited broad downregulation of migration, translation, and antiviral pathways in both CD4^+^ and CD8^+^ T cells, with limited enrichment of differentiation and antigen-presentation programs relative to TPs and HDs. These associations point to a restrained yet balanced immune profile in LTNPs, although causality cannot be inferred from this cross-sectional study.

Building on these global transcriptomic differences, we next examined specific T-cell subsets. We observed a higher percentage of CD8^+^ Effector-GNLY cells in TPs, consistent with prior reports associating this subset with poorer immune restoration (30, 40). Although naive-cell proportions may have biomarker value, further prospective validation is required. Similar findings in viremic non-progressors have linked a higher initial T-cell reservoir to CD4^+^ T-cell recovery and mitigation of immune exhaustion (32, 41), aligning with the lower naive-cell proportions we observed in TPs (30). Notably, we did not observe a discrete exhausted CD8^+^ cluster; instead, exhaustion-related differences were inferred from gene set expression levels, which should be interpreted as transcriptomic associations rather than functional evidence (42). These subset-level observations prompt further exploration of broader pathway patterns.

GO enrichment analysis revealed a consistent pattern of broad downregulation in LTNPs, spanning migration, translation, and antiviral/immune-activation pathways across both CD4^+^ and CD8^+^ T cells. These attenuated programs suggest a restrained transcriptional state that avoids excessive activation yet still preserves immune responsiveness through limited enrichment of differentiation and antigen-presentation signatures. It is well established that heightened activation can be associated with metabolic stress. In line with these enrichment patterns, CD8^+^ T cells in LTNPs displayed lower expression of biosynthetic and translation programs (e.g., ribosome biogenesis and mitochondrial translation), consistent with a restrained metabolic state. Additionally, CD4^+^ T cells showed higher JUNB/JUND and lower GIMAP4/7 expression, potentially reflecting shifts in activation- and apoptosis-related programs (43). These observations raise hypotheses linking metabolic tone to functional durability that warrant future investigation. Additionally, hdWGCNA analysis highlighted network-level shifts in LTNPs, with innate/myeloid modules increased and B-cell, NK, and platelet modules reduced. These patterns paralleled lower B-cell percentages, reduced BAFF-related expression (Fig. S8), and attenuated IFN-alpha responses with diminished interferon-stimulated gene expression, collectively suggesting a globally restrained immune network in LTNPs, the functional significance of which remains to be defined (32).

Spontaneous vRNA-expressing reservoirs have been detected in many ART-suppressed individuals and can produce viral RNA and proteins that drive inflammation (44). We observed rare HIV RNA^+^ cells using a conservative pipeline, with three cells in LTNP (CD4^+^ naïve/central memory) and 33 in TPs distributed across several subsets (CD4^+^ T and CD8^+^ T cells, NK cells, B cells, and monocytes). Given the known limitations of non-targeted 10× 3′ PBMC assays and the absence of positives in uninfected controls, we report these findings descriptively and do not infer productive infection from RNA detected in non-CD4^+^ compartments. Thus, the apparent detection of HIV RNA^+^ cells in CD8^+^ T and B cells requires further validation. We also observed a lower proportion of γδ T cells in LTNPs than in TPs and healthy donors, whose biological relevance of which warrants further study (32, 45).

Our study has several limitations. The relatively small sample size and cross-sectional design limited longitudinal interpretation, and all findings remain correlative. For certain rare subsets (e.g., CD4^+^ effector memory and NKT-like), limited cell numbers per donor may reduce the statistical robustness (35). Previous evaluations of single-cell study design have highlighted similar concerns (46); however, donor-level reanalysis yielded consistent trends, supporting the robustness of our conclusions. Because plasma HIV-1 RNA levels overlapped between groups, we did not evaluate associations between viral load and expression-based scores. Detection of HIV RNA^+^ cells in droplet-based 10× 3′ PBMC assays is inherently limited by poly(A) capture bias, and targeted enrichment approaches were not used here (20). Finally, untreated LTNPs could not be recruited for further validation under current test-and-treat policies. Nevertheless, our transcriptomic trends are directionally consistent with prior T-cell studies, underscoring their biological relevance.

In this cross-sectional cohort study, we refined the transcriptional landscape of non-progression in chronic HIV infection. Although these observations represent associations rather than causal mechanisms, they provide testable hypotheses and a framework for future mechanistic and functional studies.

## MATERIALS AND METHODS

### Patients

A total of 12 participants were recruited, including four long-term non-progressors (LTNPs), four typical progressors (TPs), and four healthy donors (HDs). LTNPs are defined as HIV-infected individuals who have remained ART-naive for more than 7 years while maintaining CD4^+^ T-cell counts consistently above 500 cells/µL during this period, regardless of plasma viral load levels (9, 11, 12, 47). TPs were defined as HIV-infected individuals whose CD4^+^ T-cell counts declined to below 500 cells/µL within 2–5 years of infection without ART, consistent with previously reported criteria (11). All participant information was obtained from the National Notifiable Disease Surveillance System (NNDSS) of the Chinese Center for Disease Control and Prevention (China CDC) and confirmed with participants at enrollment. According to the national program, ART initiation requires registration, which is recorded in this system. The absence of ART initiation or dispensing records for all LTNP and TP participants was confirmed, establishing their ART-naive status at sampling. For each participant, longitudinal CD4^+^ T-cell counts prior to ART initiation were abstracted from clinical records, and per-donor trajectories are shown in Fig. S1. Healthy donors were HIV-seronegative adults confirmed by anti-HIV antibody testing, with no major comorbidities and in good health. Inclusion and exclusion criteria are provided in Table S4.

### Preparation of single-cell suspensions

Peripheral blood mononuclear cells (PBMCs) were initially isolated by Ficoll-Hypaque density gradient centrifugation from EDTA-anticoagulated blood collected at enrollment from eight individuals with chronic HIV infection and four HDs. All cell separations were performed according to the manufacturer’s instructions. Cell concentration was determined using a hemocytometer, and cell viability was assessed by trypan blue staining (cell viability >80%). An appropriate volume of pre-cooled wash buffer was added to achieve a final cell concentration of 700–1,200 cells/μL (7 × 10⁵−1.2 × 10⁶ cells/mL) and samples were immediately proceed the 10× Genomics Single Cell workflow.

### Library construction and sequencing

Cellular suspensions were processed using the 10× Genomics Chromium Single-cell instrument, which produced single-cell Gel Bead-In-Emulsion (GEMs). Libraries were constructed using Chromium Next GEM Single Cell 3′ Expression Kit (10× Genomics) according to the manufacturer’s protocol. Upon gel bead dissolution within GEMs, primers including an Illumina R1 sequence, a 16 nt 10× Barcode, a 10 nt Unique Molecular Identifier (UMI), and a poly-dT sequence were released, initiating reverse transcription of barcoded, full-length cDNAs from polyadenylated mRNA. After purifying the cDNA, libraries were prepared by PCR amplification, and sequencing adapters (P5, P7) and sample indices were added. High-throughput sequencing was performed on the Illumina NovaSeq X Plus platform. The resulting Single Cell 3′ libraries contained Illumina paired-end constructs, with the 10× barcode and UMI encoded in Read 1 and the cDNA fragment sequenced in Read 2. The i7 index read was used to incorporate sample index sequences.

### scRNA-seq data processing

After converting the BCL files to FASTQ files, raw reads were processed with Cell Ranger version 3.1.0 for alignment to the human genome (GRCh38), cell barcode assignment, and UMI counting. In R software, the count matrix was read to create a Seurat object using the Seurat package (version 5.1.0) (48). We retained cells with ≥200 detected genes (min.features = 200) and retained genes detected in ≥3 cells (min.cells = 3). Low-quality cells were further filtered to retain those with nFeature_RNA < 4,000 and percent.mt <20%. In total, 159,493 single-cell transcriptomes were analyzed. Gene expression was normalized with SCTransform, followed by reference mapping to an integrated atlas (31) and UMAP visualization. CD4^+^ and CD8^+^ T cells were subsetted and further filtered to retain cells with nFeature_RNA < 2,000, percent.mt <15%, and percent.Hb <0.5%. We re-normalized with SCTransform, performed PCA, and applied Harmony for batch correction. Unsupervised graph-based clustering (FindNeighbors/FindClusters) was performed on the Harmony embedding (resolution = 0.2 for CD4^+^ T cells; 0.15 for CD8^+^ T cells). t-SNE was used for visualization only, using the top 25 (CD4^+^) or 20 (CD8^+^) Harmony dimensions.

### Cluster marker identification and cell-type annotation

For visualization purposes, non-linear dimensionality reduction (t-SNE) was performed on CD4^+^ and CD8^+^ T-cell subsets to project cells into a two-dimensional space. FindAllMarkers (Seurat) was used to identify cluster markers. Clusters were then classified and annotated based on canonical markers and differential expression patterns, following prior reports (25, 30).

### DEG identification and functional enrichment

Differentially expressed genes (DEGs) were identified using FindMarkers (Seurat) with test. use = "t." Adjusted p-values were calculated using Bonferroni correction. DEGs were filtered by |log₂ fold change| ≥ 0.5 and FDR ≤ 0.05. Enrichment analysis for DEG functions was performed using the clusterProfiler (version 4.8.3) and org.Hs.eg.db (version 3.17.0) R packages.

### Calculation of cellular state scores

To evaluate gene set expression in individual cells, cell state scores were computed to represent the average expression levels of genes within each set for each cell. For a given cell χ and gene set k (denoted as Gk), the cell score SCk(χ) measures the relative expression of Gχ in cell χ. This score is computed as the difference between the average relative expression (Er) of genes in Gk and the average relative expression of a control gene set (Gkcont): SCk(c) = mean (Er[Gκ,c]) − mean (Er [Gκcont, c]). The control gene set was chosen randomly from bins based on overall expression levels, ensuring it had similar or higher expression distributions compared with the gene set of interest. This procedure was implemented using the AddModuleScore function in Seurat with its default parameters. As in prior HIV scRNA-seq studies (30), the scores were calculated for various biological processes using specific gene sets: RESPONSE TO INTERFERON ALPHA (GO:0035455), INFLAMMATORY RESPONSE (GO:0006954), APOPTOTIC SIGNALING PATHWAY (GO:0097190), T CELL ACTIVATION (GO:0042110), LEUKOCYTE MIGRATION (GO:0050900), as well as four established naive markers (CCR7, TCF7, LEF1, and SELL), 12 genes associated with cytotoxicity (PRF1, IFNG, GNLY, NKG7, GZMB, GZMA, GZMH, KLRK1, KLRB1, KLRD1, CTSW, and CST7), and seven markers of cellular exhaustion (LAG3, TIGIT, PDCD1, CTLA4, HAVCR2, TOX, and CD244). These gene sets were utilized to derive scores related to IFN-α response, inflammatory response, apoptosis, activation, migration, naive state, cytotoxicity, and exhaustion. Throughout the manuscript, all “scores” denote per-cell gene-set expression scores (AddModuleScore) derived from the specified multi-gene sets; they reflect transcriptomic signatures and are not single-gene readouts or direct functional assays.

### Identifying HIV-1 RNA^+^ cells

To identify HIV RNA^+^ cells, raw scRNA-seq reads were aligned using BWA-MEM (version 0.7.17) (49) to a combined reference consisting of the human genome (GRCh38.p14) and HIV-1 reference sequences obtained from the NCBI Virus database. Alignments to the HIV genome with aligned length >100 bp were retained, and potential false positives were removed by BLAST (version 2.15.0^+^ ) (50). Cell barcodes and UMIs from Cell Ranger were then used to assign viral reads to individual cells and compute per-cell viral UMI counts. Cells with ≥1 HIV-1 read were classified as HIV RNA^+^. No HIV-1 reads were detected in HD control samples.

### High-dimensional WGCNA (hdWGCNA) analysis

The hdWGCNA package (version 0.2.2) developed by Morabito et al. was used for weighted gene co-expression network analysis (WGCNA) on single-cell data (51). Networks were constructed following the standard package workflow. Soft-threshold power selection was performed using TestSoftPowers, and a power of 4 was chosen to achieve an adequate scale-free topology fit (see Results, Fig. 5A). Modules were identified from the topological overlap matrix (TOM)–based hierarchical clustering and merged to yield nine co-expression modules (Fig. 5B through D). Module eigengenes were computed for inter-module correlation analyses (Fig. 5C) and downstream comparisons. Hub genes were ranked by module eigengene connectivity (kME), and the top 25 genes per module were used for GO enrichment and module labeling (Fig. S4). Analyses followed the official hdWGCNA tutorial at https://smorabit.github.io/hdWGCNA/articles/basic_tutorial.html.

## Data Availability

The raw sequence data reported in this study have been deposited in the Genome Sequence Archive in National Genomics Data Center, China National Center for Bioinformation / Beijing Institute of Genomics, Chinese Academy of Sciences (GSA Human: HRA011754), and are publicly accessible at https://ngdc.cncb.ac.cn/search/specific?db=hra&q=HRA011754.

## SUPPLEMENTAL MATERIAL

Figures S1 and S2 jvi.01597-25-s0001.docx Longitudinal trajectories of CD4^+^ T cell counts in LTNPs and TPs prior to ART initiation, and identification of main cell types.

Figures S3 to S5 jvi.01597-25-s0002.docx Marker gene expression for CD4^+^ and CD8^+^ T-cell clusters and transcriptomic profiling of CD4^+^ and CD8^+^ T cells across the three conditions. and

Figure S6 jvi.01597-25-s0003.docx hdWGCNA analysis of scRNA-seq data.

Figures S7 and S8 jvi.01597-25-s0004.docx Gene Ontology (GO) enrichment analysis of modules identified through hdWGCNA and BAFF expression in dendritic cells (DCs).

Table S1 jvi.01597-25-s0005.xlsx Characteristics of HIV-infected individuals and healthy donors.

Table S2 jvi.01597-25-s0006.xlsx Per-donor breakdown of immune cell numbers across major subsets and T-cell subpopulations.

Table S3 jvi.01597-25-s0007.xlsx Information on cells with HIV transcripts detected per donor.

Table S4 jvi.01597-25-s0008.xlsx Inclusion and exclusion criteria for study participants.

ASM does not own the copyrights to Supplemental Material that may be linked to, or accessed through, an article. The authors have granted ASM a non-exclusive, world-wide license to publish the Supplemental Material files. Please contact the corresponding author directly for reuse.
