# Supplementary material for: Single-cell transcriptome profiling reveals immunological fitness of HIV long-term non-progressors
Source: J Virol. 2025 Nov 24;99(12):e01597-25. doi: 10.1128/jvi.01597-25 (PMC12724274; doi:10.1128/jvi.01597-25)
Supplement: Figures S1 and S2 — Longitudinal trajectories of CD4+ T cell counts in LTNPs and TPs prior to ART initiation, and identification of main cell types. [file jvi.01597-25-s0001.docx]

**Supplemental Figures**


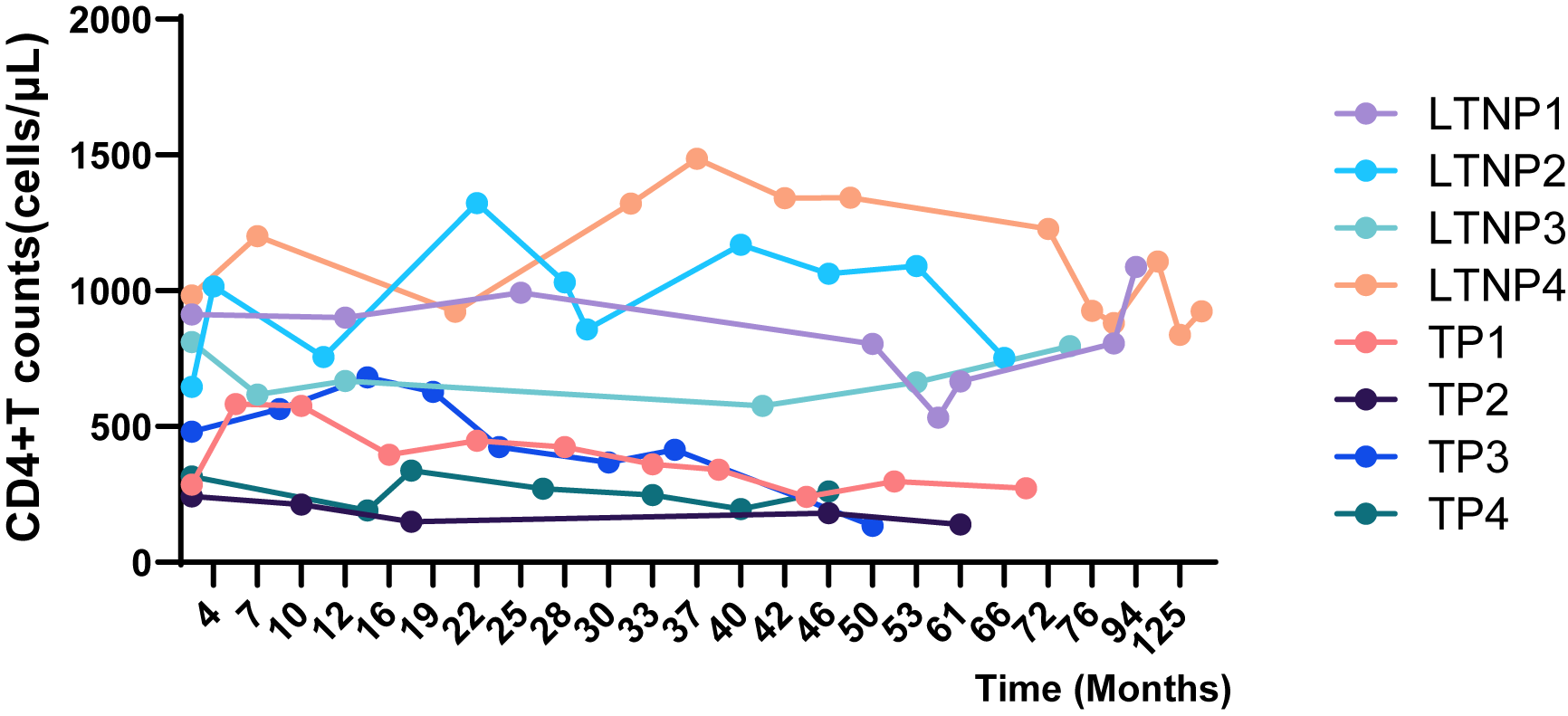


**Fig S1. Longitudinal** trajectories **of CD4⁺ T cell counts in LTNPs and TPs prior to ART initiation**

Longitudinal changes of CD4⁺ T cell counts in Long-Term Non-Progressors (LTNPs) and Typical Progressors (TPs) prior to antiretroviral therapy (ART). The x-axis denotes the number of months since each donor’s first available CD4 measurement, reflecting the follow-up interval rather than the time of HIV diagnosis.


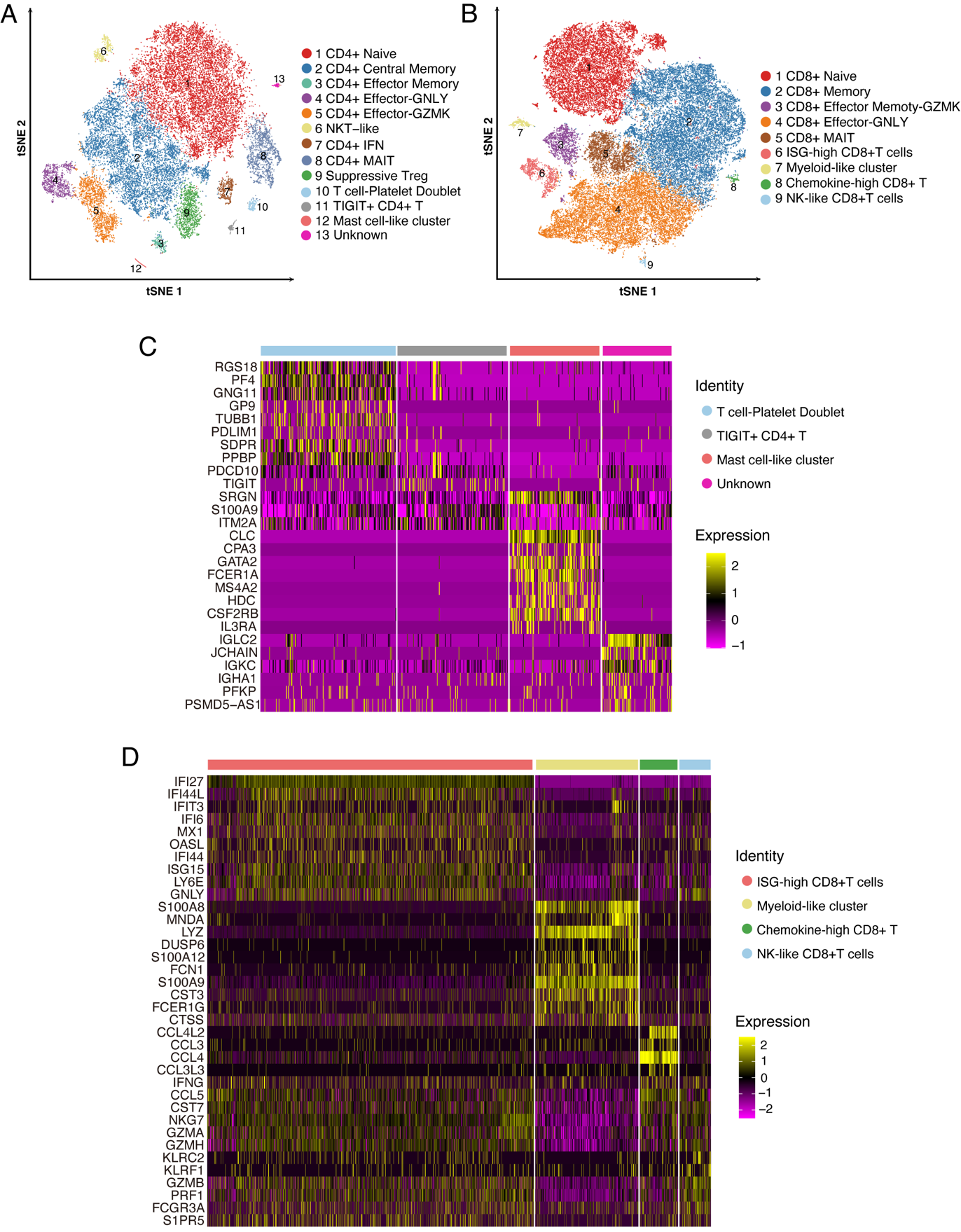


**Fig S2. Identification of main cell types**

**(A)** t-SNE projection of 26,209 CD4⁺ T cells from HD (n = 4), LTNP (n = 4), and TP (n = 4) samples, showing 13 transcriptionally distinct clusters. Each dot represents a single cell, colored by cluster annotation. **(B)** t-SNE projection of 44,817 CD8⁺ T cells from HD (n = 4), LTNP (n = 4), and TP (n = 4) samples, revealing 9 major clusters. Each dot represents a single cell, colored by cluster annotation. **(C)** Heatmap showing representative top differentially expressed genes (DEGs) across the indicated CD4⁺ T-cell subsets. **(D)** Heatmap showing representative top DEGs across the indicated CD8⁺ T-cell subsets. Low-abundance subsets are displayed for visualization only and were excluded from downstream analyses.
