# Supplementary material for: Single-cell transcriptome profiling reveals immunological fitness of HIV long-term non-progressors
Source: J Virol. 2025 Nov 24;99(12):e01597-25. doi: 10.1128/jvi.01597-25 (PMC12724274; doi:10.1128/jvi.01597-25)
Supplement: Figures S3 to S5 — Marker gene expression for CD4+ and CD8+ T-cell clusters and transcriptomic profiling of CD4+ and CD8+ T cells across the three conditions. and [file jvi.01597-25-s0002.docx]

**Supplemental Figures**


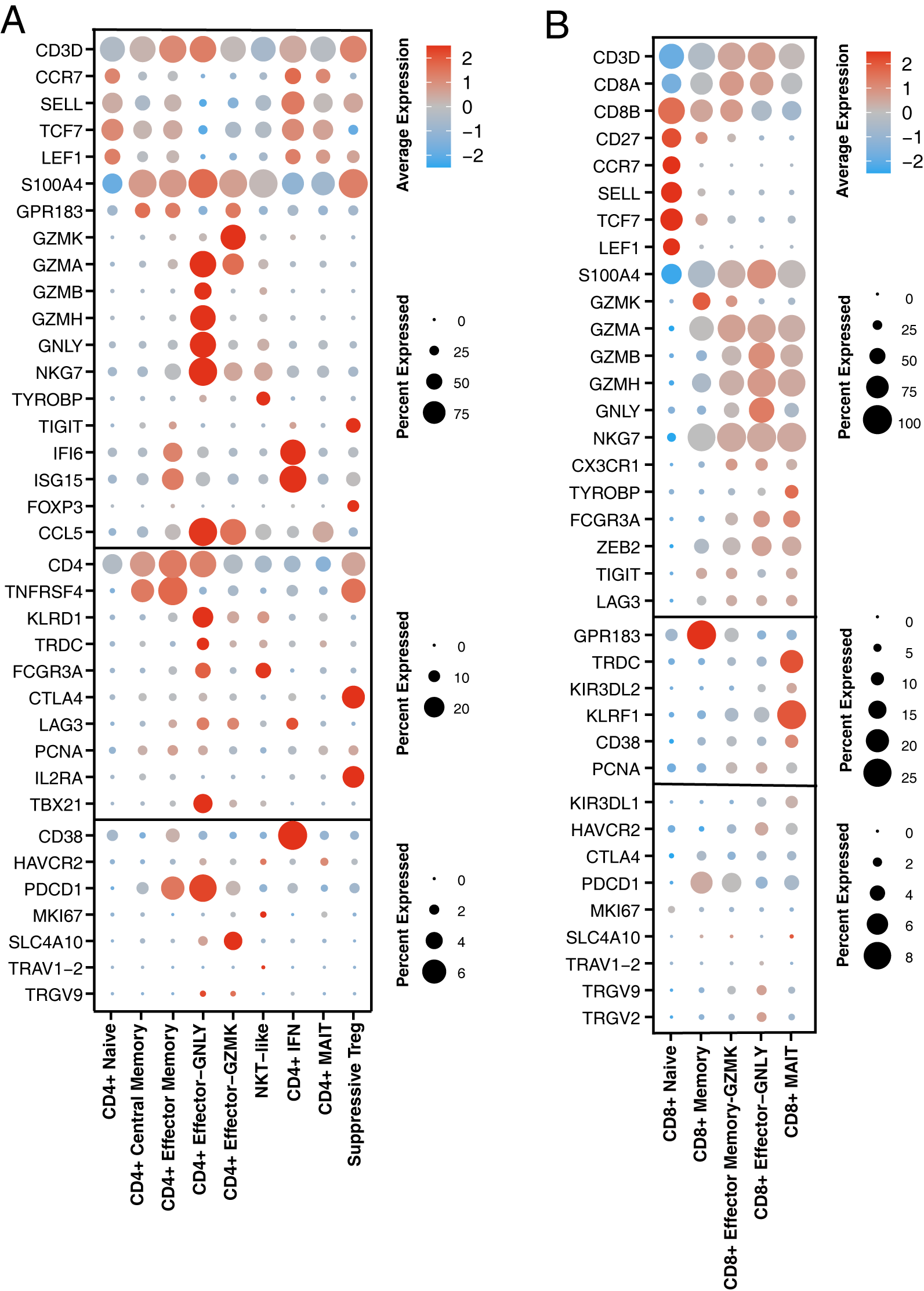


**Fig S3. Marker gene expression for CD4⁺ and CD8⁺ T-cell clusters.**

**(A)** Dot plots showing the expression of selected marker genes across the 9 CD4⁺ T-cell clusters (columns correspond to clusters colored as in Fig. 1D. **(B)** Dot plots showing the expression of selected marker genes across the 5 CD8⁺ T-cell clusters (columns correspond to clusters colored as in Fig. 1E. Rows indicate marker genes. Dot size represents the percentage of cells expressing each gene, and color intensity indicates scaled expression (z-score).


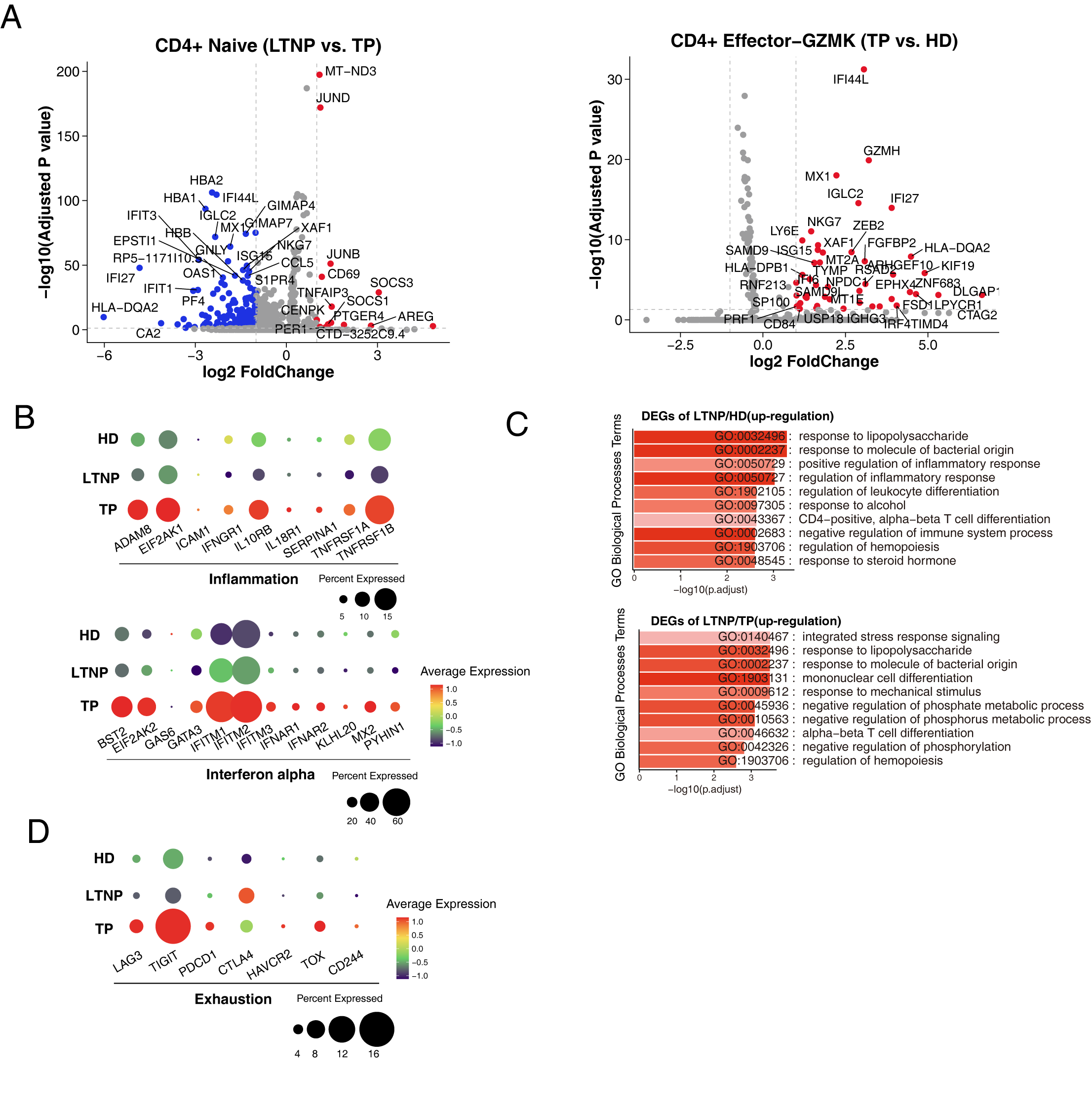


**Fig S4. Transcriptomic profiling of CD4+ T cells across the three conditions.**

**(A)** Volcano plots display differentially expressed genes (DEGs) for CD4⁺ T-cell subsets across the three groups, filtered by adjusted p ≤ 0.05 and average log₂(fold change) ≥ 0.25. Only subset pairs with significant gene-expression differences, as defined in Fig. 1F, are included. **(B)** Dot plot showing expression of inflammation-related genes (*Top*) and interferon alpha-related genes (*bottom*) across the three conditions. Circle size represents the percentage of expressing cells, and color intensity indicates scaled expression levels. **(C)** GO enrichment analysis of upregulated DEGs in LTNPs versus HDs (top) and in LTNPs versus TPs (bottom). GO terms are labeled with names and ids and ranked by −log10 (P) value. A brighter color indicates a higher number of genes. The top 10 enriched GO terms of the DEGs in CD8+T cell between LTNP/TP and HD are shown with adjusted p value≤0·05 and average log2 (fold change)≥0·5. **(D)** Similar to **(B)**, illustrating the expression of exhaustion-associated genes across the three conditions.

**
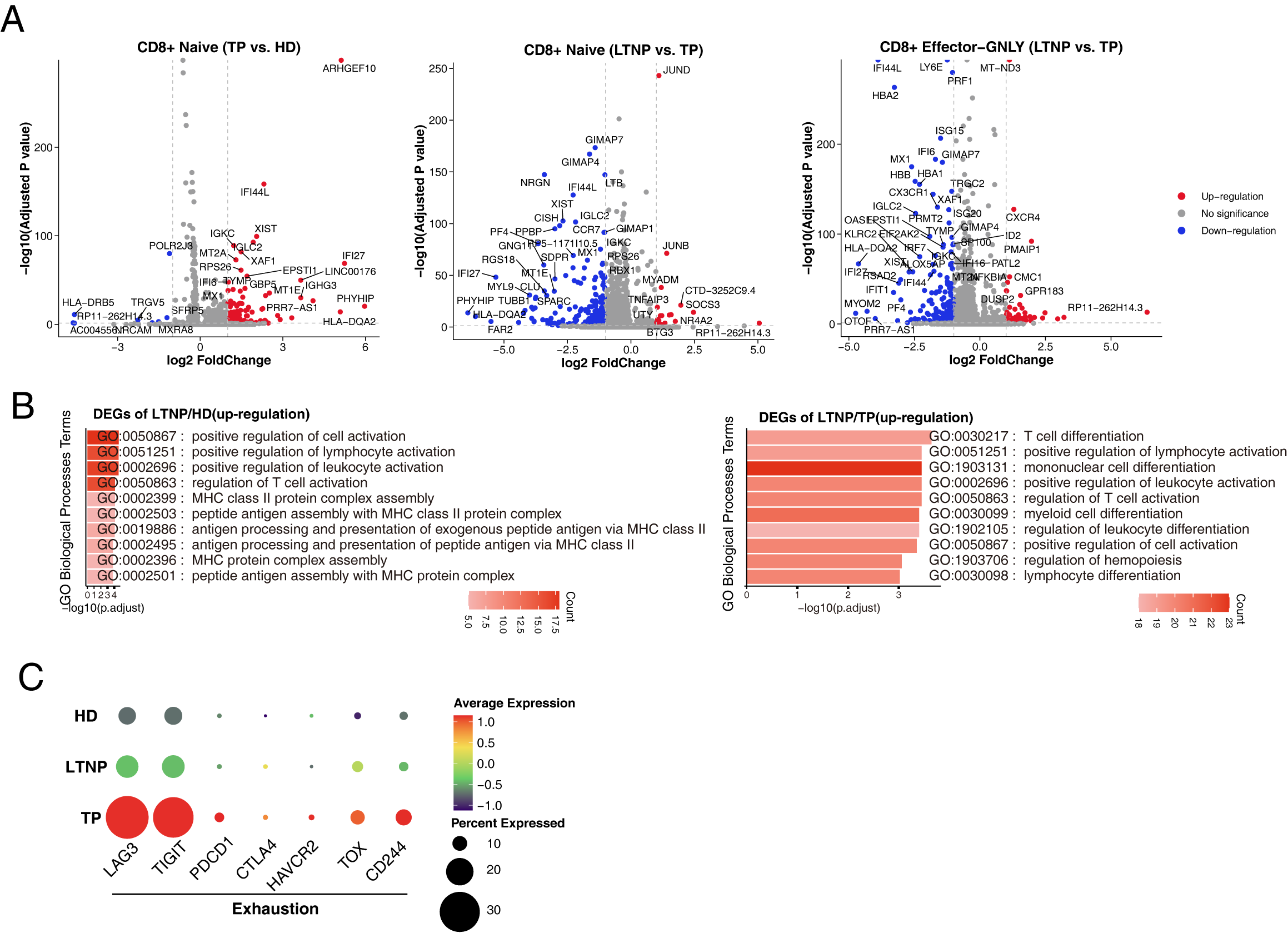
**

**Fig S5. Transcriptomic profiling of CD8^+^ T cells across the three conditions.**

**(A)** Volcano plots showing differentially expressed genes (DEGs) for CD8⁺ T-cell subsets across the three groups, filtered by adjusted p ≤ 0.05 and average log₂(fold change) ≥ 0.25. Only subset pairs with significant gene-expression differences, as defined in Fig. 1G, are shown. **(B)** GO enrichment of upregulated DEGs in LTNPs compared with healthy donors (*left*), and in LTNPs compared with TPs*(right*). GO terms are labeled with name and id and sorted by −log10 (P) value. A brighter color indicates a higher number of genes. The top 10 enriched GO terms of the DEGs in CD8^+^ T cell between LTNP/TP and HD are shown with adjusted p value≤0·05 and average log2 (fold change)≥0·5.  **(C)** Similar to Fig S4D, but displaying exhaustion-associated gene signatures in CD8⁺ T cells.
