# Supplementary material for: Single-cell transcriptome profiling reveals immunological fitness of HIV long-term non-progressors
Source: J Virol. 2025 Nov 24;99(12):e01597-25. doi: 10.1128/jvi.01597-25 (PMC12724274; doi:10.1128/jvi.01597-25)
Supplement: Figure S6 — hdWGCNA analysis of scRNA-seq data. [file jvi.01597-25-s0003.docx]

**Supplemental Figures**


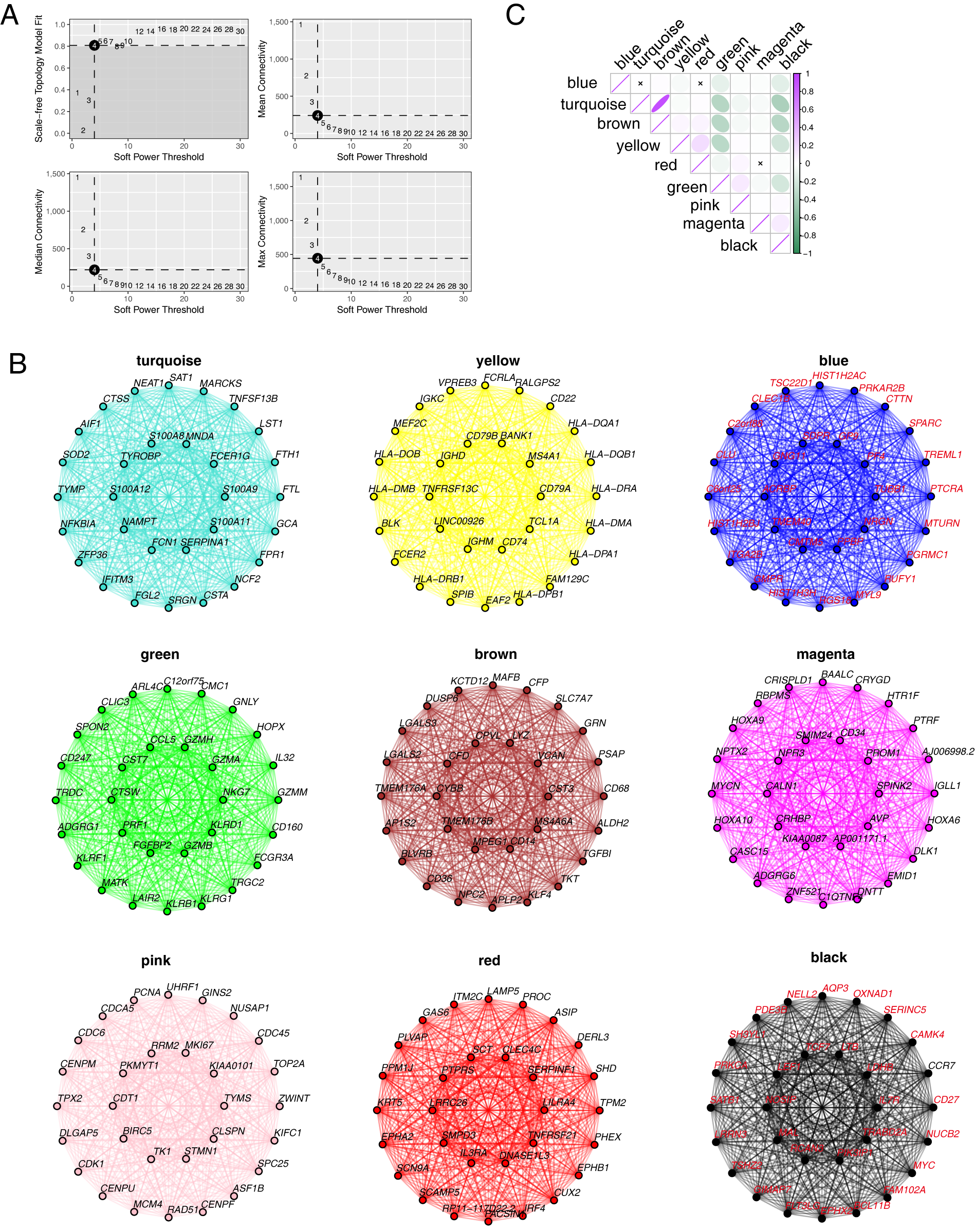


**Fig S6.** **hdWGCNA analysis of scRNA-seq data.**

**(A)** Selection of the optimal soft-thresholding. **(B)** Matrix plot showing the inter-module relationships based on module eigengene correlation. **(C)** Visualization of hub genes within the nine modules identified by hdWGCNA. Nodes represent individual genes, with connectivity highlighted; the top 10 genes in each module are prominently labeled. Modules are color-coded as turquoise, yellow, blue, red, brown, green, and black, corresponding to their classifications in the co-expression network.
