# Supplementary material for: Single-cell transcriptome profiling reveals immunological fitness of HIV long-term non-progressors
Source: J Virol. 2025 Nov 24;99(12):e01597-25. doi: 10.1128/jvi.01597-25 (PMC12724274; doi:10.1128/jvi.01597-25)
Supplement: Figures S7 and S8 — Gene Ontology (GO) enrichment analysis of modules identified through hdWGCNA and BAFF expression in dendritic cells (DCs). [file jvi.01597-25-s0004.docx]

**Supplemental Figures**


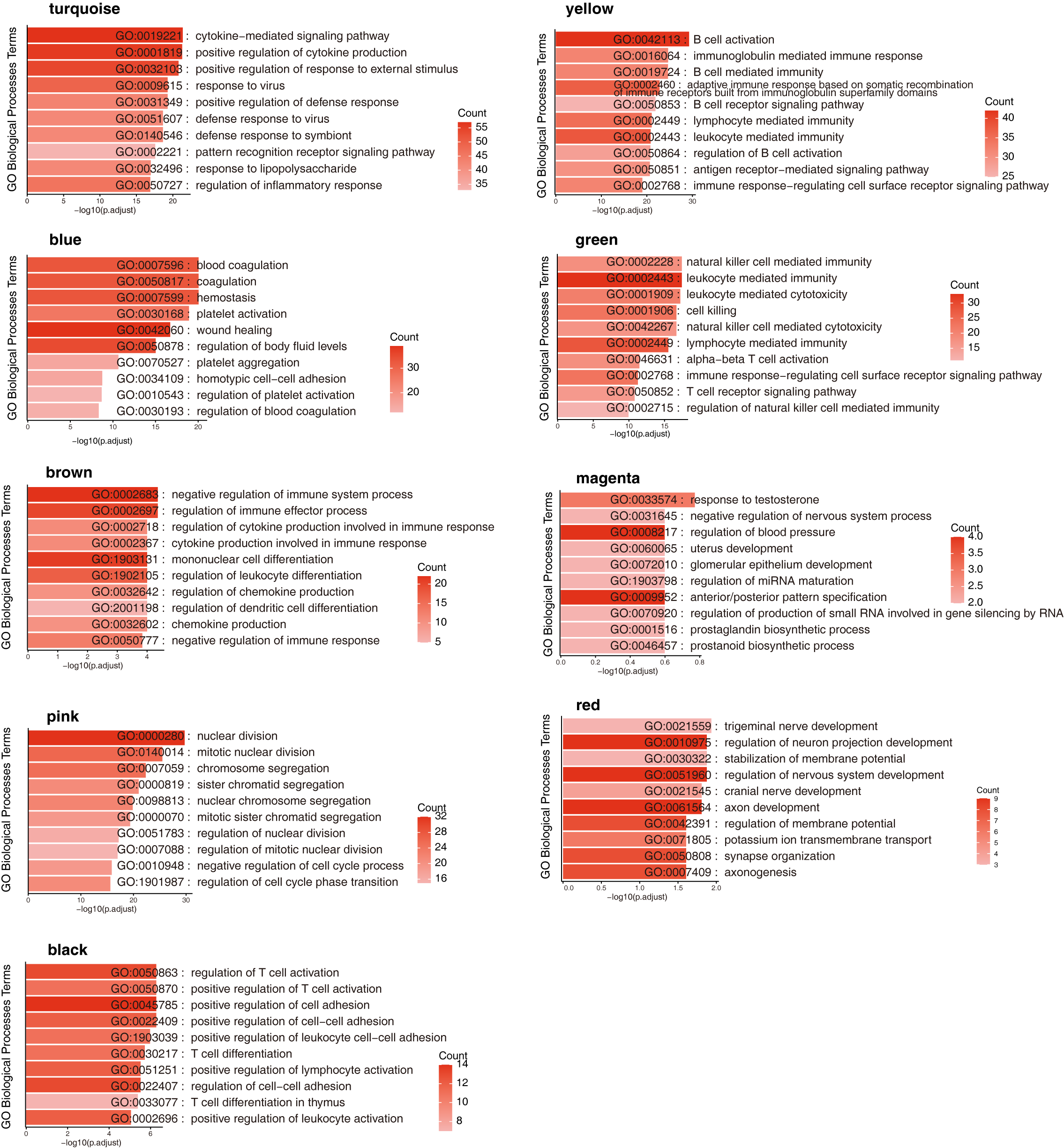


**Fig S7. Gene Ontology (GO) enrichment analysis of modules identified through hdWGCNA.**

GO biological process terms enriched in genes from each module are shown and categorized by module color (turquoise, yellow, blue, red, brown, green, and black). The x-axis represents enrichment significance as –log₁₀ (adjusted p-value), and the y-axis displays the top enriched GO terms for each module. Bar size indicates the number of genes contributing to each term.

**
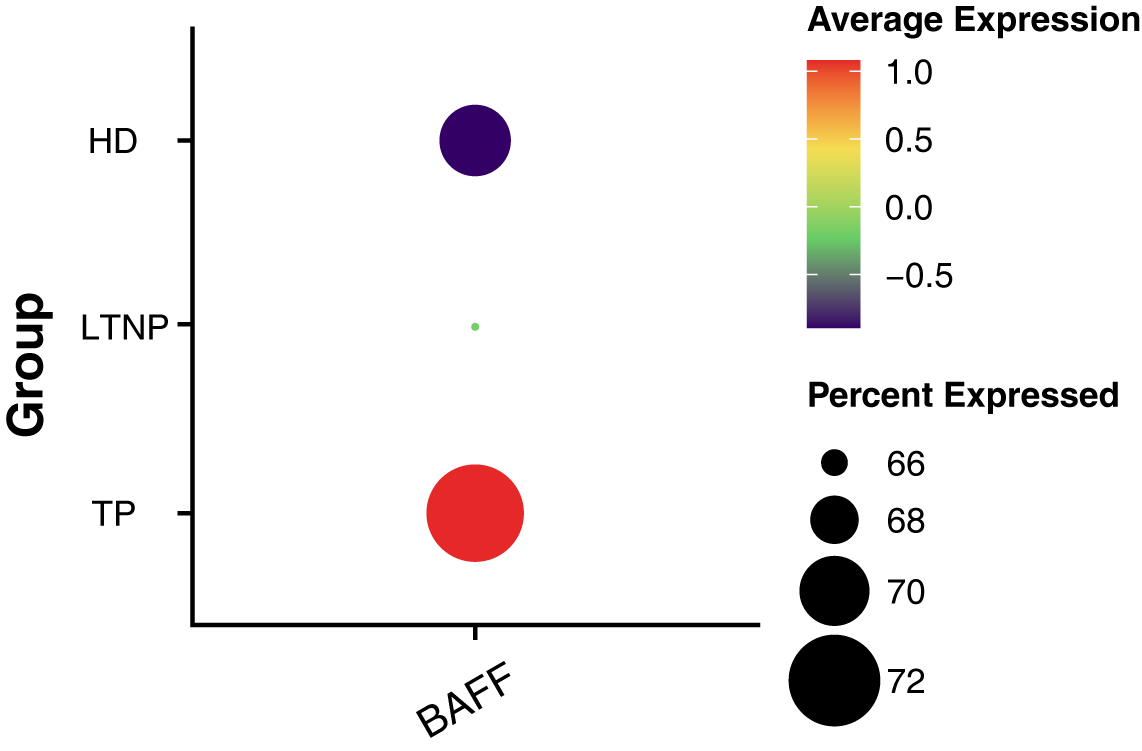
**

**Fig S8. BAFF expression in dendritic cells (DCs).**

BAFF expression levels in dendritic cells are shown across all donors and conditions.
